# Primary Anorectal Mucosal Melanoma: A Unique Presentation of Mucosal Melanomas

**DOI:** 10.7759/cureus.70100

**Published:** 2024-09-24

**Authors:** Fatima Rezzoug, Jihane Derfoufi, Ouissam Al Jarroudi, Sami Aziz Brahmi, Said Afqir

**Affiliations:** 1 Medical Oncology, Mohammed VI University Hospital, Oujda, MAR; 2 Faculty of Medicine and Pharmacy, Mohammed First University, Oujda, MAR

**Keywords:** anorectal malignancies, anorectal melanoma, case report, malignant melanoma, mucosal melanoma, treatment

## Abstract

Anorectal mucosal melanoma (AMM) is a rare and highly aggressive malignancy. It frequently presents with nonspecific symptoms, often resulting in delayed diagnosis and poor prognosis. This report describes the case of a 60-year-old male who presented with a painful para-anal papule that progressed to a fistula. Histopathological and immunohistochemical analyses confirmed AMM. Imaging revealed a locally advanced tumor without distant metastasis. Due to the locally advanced nature of the disease, a multidisciplinary team recommended neoadjuvant radiotherapy. This case highlights the diagnostic and therapeutic challenges associated with AMM and emphasizes the importance of a tailored, multidisciplinary approach. Surgical resection remains the cornerstone of treatment, with neoadjuvant therapy potentially improving surgical outcomes in advanced cases.

## Introduction

Melanoma is a neoplasm that arises from melanocytes, cells derived from embryonic neural crest cells found in the skin, eyes, and mucous membranes [1]. Most melanomas are cutaneous, but mucosal melanomas account for 1.4% of cases and can occur in various body regions [2]. Anorectal mucosal melanoma (AMM) is a rare subtype, constituting 0.4%-1.6% of all malignant melanomas and about 4% of anal cancers [2,3].

Anorectal melanoma (AM) is an uncommon and aggressive cancer originating from mucosal melanocytes [4]. This illness is more frequently seen in females and often manifests in the fifth or sixth decade of life [4]. Given its anatomical position and nonspecific symptoms, diagnosing AMM in its early stages is difficult, explaining its low survival rates. Consequently, it usually takes four to six months for symptoms to be recognized before diagnosis, which means the diagnosis is often delayed [5]. Histology and immunohistochemistry are the ultimate diagnostic procedures, although computed tomography (CT) and magnetic resonance imaging (MRI) are utilized to help in the staging of AMM [6].

Unfortunately, most cases of AMs are diagnosed with distant metastases already present, leading to a poor prognosis [4]. The five-year survival rate is lower than 20% and the median post-treatment survival time is less than two years [5]. Despite aggressive treatment, the prognosis remains dismal, and AM has a worse outcome compared to the cutaneous form [5]. To date, there is no established standard treatment for AMM, and often, radical surgery is the main method used [6]. Here, we report a rare case of primary AM in a 60-year-old male.

## Case presentation

A 60-year-old male, a heavy smoker with no personal or family history of malignancy, presented with a painful para-anal papule that had been present for several months. The lesion gradually increased in size and eventually developed into a fistula with purulent discharge. No weight loss was registered.

The general examination was unremarkable, finding the patient in good physical condition (PS = 1). The abdominal examination was normal, and there was no clinical involvement of the inguinal nodes or any suspicious cutaneous or extra-digestive lesions. Proctological examination revealed a brownish ulcerative-vegetating mass, 3 cm in diameter, located external to the anal verge and hard on palpation.

**Figure 1:**
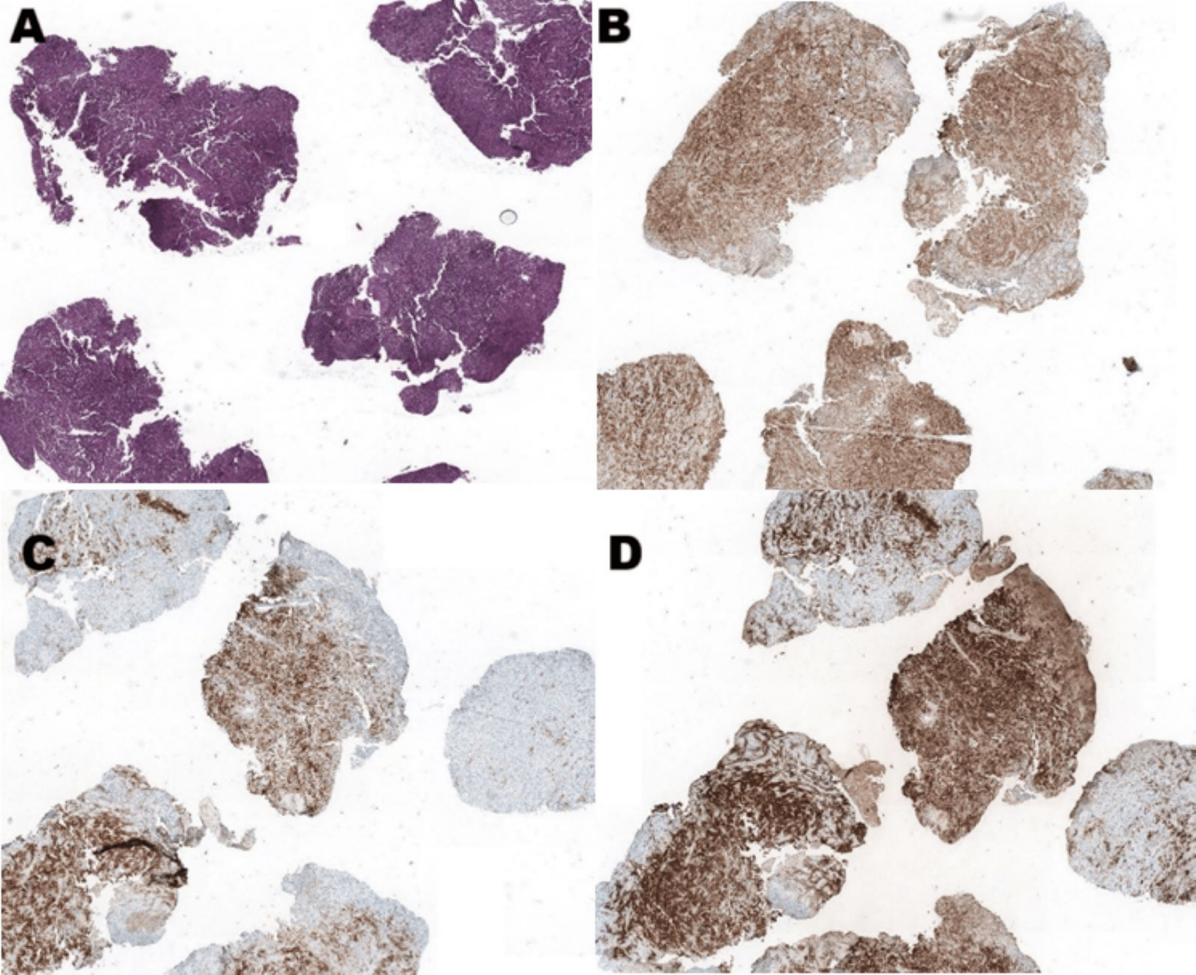
Histopathological and immunohistochemical examination of the tumor. (A) Histopathological evaluation demonstrating malignant melanoma (hematoxylin and eosin, ×4). (B) immunohistochemistry staining showing positivity for S-100 protein. (C) immunohistochemistry staining showing positivity for Melan A. (D) immunohistochemistry staining showing positivity for human melanoma black-45.

Initially, a biopsy of the anal lesion was performed, which revealed malignant melanoma. The immunohistochemical staining was positive for S-100 protein, Melan, and human melanoma black-45 (HMB-45), but negative for anti-AE1/AE3 (Figure 1).

A more complete workup was then performed to identify the tumor’s location and distant dissemination to choose the most effective treatment.

**Figure 2:**
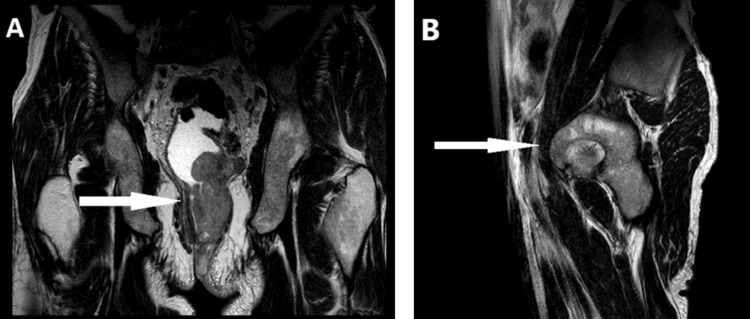
Pelvic magnetic resonance imaging scan. (A, B): coronal and sagittal images showing the presence of a circumferential, irregular, and asymmetrical tumor mostly on the left side of the lower rectum and anal area. The tumor was 51 mm thick and stretched over 10 cm.

Initially, a pelvic MRI showed the presence of a circumferential, irregular, and asymmetrical tumor mostly on the left side of the lower rectum and anal area. The tumor was 51 mm thick and stretched over 10 cm. It extended significantly into the anterolateral left mesorectum, infiltrating the right internal sphincter, the internal and external sphincters, the puborectalis muscle, and the levator ani muscle, with only slight infiltration of the left ischiorectal and ischioanal tissue (tumor classified as T4) (Figure 2). A full CT scan did not show any distant secondary lesions.

The treatment strategy was determined by a multidisciplinary team assessment involving surgeons, radiotherapists, and oncologists. The team decided to initiate neoadjuvant radiotherapy due to the locally advanced nature of the disease.

## Discussion

AMMs are considered to be uncommon. Here, we present the case of a 60-year-old man with AMM, who initially presented with a painful para-anal papule that evolved into a purulent fistula.

Melanomas develop in areas of the body that contain melanocytes, such as the skin, eyes, nasal cavity, oropharynx, vagina, urinary tract, rectum, and anus [1]. Mucosal melanomas are rare tumors, accounting for 1.4% of all melanoma cases [2]. The anorectum is the third most frequent location for mucosal melanomas, following the head and neck and the female genital tract [7]. AMM is a very rare and aggressive form of melanoma, accounting for only 0.4%-1.6% of all malignant melanomas and approximately 4% of anal cancers [2,3]. People of all ages can develop AMM, but those in their 60s and 70s are more likely to have it [4]. However, reports of cases have also included young individuals, such as 11-year-olds and 19-year-olds [8]. Women are 1.6-2.3 times more likely than men to develop AMM [4]. Additionally, Caucasians have twice the incidence rate of AMM compared to African Americans [5]. Here, we report a case of primary AM in a 60-year-old male.

Patients with AMM may experience a variety of symptoms, including pain, rectal bleeding, rectal mass, proctalgia, pruritus, and changes in bowel habits [6]. AMM may sometimes present as hemorrhoids, polyps, or skin tags [6]. These vague symptoms often imitate those of other anorectal disorders, resulting in delays in detection [6]. Due to its generic nature, people often misdiagnose AMM as other anal diseases such as complicated hemorrhoids [6]. The median time from the onset of symptoms to diagnosis confirmation varies between four and six months [5]. Overall, 8%-16% of patients receive a diagnosis following a hemorrhoidectomy [9]. Our patient presented with a painful para-anal papule that had been present for several months.

Upon diagnosis, around 70% of patients show signs of metastases. These metastatic occurrences are commonly found in the mesorectal or inguinal lymph nodes, similar to other carcinomas in this area. Hematogenous metastases typically target the liver, lungs, brain, and bones [10].

Diagnosing AMM typically involves conducting biopsies, which is considered the gold-standard approach. Proctoscopy, or colonoscopic biopsies, are usually performed to collect tissue samples for analysis. Colonoscopy aids in determining various characteristics of the lesion, such as its site, surface, color, and invasion of the dentate line. Histology and immunohistochemistry are crucial for diagnosing AMM. Histopathological examinations often reveal high pleomorphism in the nucleus, spindle-shaped epithelioid, and melanin granules [1]. These features can complicate the differential diagnosis with other tumors such as sarcomas, gastrointestinal stromal tumors (GIST), and undifferentiated carcinomas [1]. Immunohistochemistry plays a pivotal role in differentially diagnosing AMM from other rare pigmented malignancies. Key immunohistochemistry markers for diagnosing malignant melanoma include HMB-45, S-100, melan A, and vimentin [6]. AMM is typically negative for carcinoembryonic antigen, cytokeratin, and epithelial membrane antigen [5]. Our patient had undergone a biopsy, and immunohistochemistry was consistent with the diagnosis of AMM.

AMM has distinct risk factors and genetic characteristics compared to cutaneous melanomas. A *c-KIT* activating mutation and a family history of the disease are risk factors [11]. Studies have shown that cutaneous melanomas are often linked to the *BRAF V600E* mutation, whereas AMMs are more frequently associated with a *c-KIT* mutation [12]. Immunological factors such as human papillomavirus and human immunodeficiency virus infections may also play a role, but the evidence is inconclusive [13]. The exact pathogenesis of the disease is not known. However, some theories suggest genetic alterations in intracellular signaling cascades, but the optimal targeting approach is unknown [14]. AMM may originate from melanocytes near the dentate line that progresses upward from the submucosa toward the rectum [15]. Other theories propose origins from Schwannian neuroblastic cells or cells of the amine-precursor uptake and decarboxylation system [10]. The higher incidence in females might be linked to the effects of estrogen on melanocyte number and melanin content [10]. Recent findings have uncovered many oncogenes that are involved in the development of melanoma, leading to the development of targeted therapies [16]. The protein c-KIT plays a crucial role in diagnosing GISTs and differentiating them from other mesenchymal tumors in the gastrointestinal tract [9]. Mucosal melanomas, including AMM, frequently exhibit *KIT* mutations, with a reported incidence of 35.5% in AMM [15]. On the other hand, the occurrence of *BRAF* and *NRAS* mutations in AMM is less frequent than in cutaneous melanomas, although *KIT* mutations are more common [13].

Following the histologic diagnosis of AMM, it is crucial to conduct a thorough assessment for metastases, as they can be present in 20%-65% of patients at the initial diagnosis [17]. This assessment involves performing thoracic, abdominal, pelvic, and brain CT scans, and considering a positron emission tomography (PET) scan. Pelvic MRI, anal endo-ultrasound, and rectoscopy help evaluate the tumor’s extent and local infiltration to guide surgical planning [17]. Imaging modalities such as abdominal ultrasound, CT scans, and MRI are essential for staging and managing patients. On MRI, AMM typically appears hyperintense on T1-weighted images and hypointense on T2-weighted images due to the paramagnetic properties of the melanotic component, although these features may be absent in amelanotic melanoma [18]. PET-CT is valuable for staging, assessing therapy response, and detecting distant metastases [6]. A complete colonoscopy is necessary to rule out synchronous lesions [6]. Our patient underwent an MRI of the abdomen and pelvis, which was consistent with a diagnosis of AMM. There was no evidence of nodal or distant metastasis on the CT scan at presentation.

Clinical and pathological data are used to stage AMM, but unlike other cancers, there is no defined pathologic staging scheme for AMM. Typically, the stage is determined based on the pattern of spread, with stage I indicating local disease, stage II indicating local disease with regional lymph nodes, and stage III indicating distant metastases [19]. The liver, lungs, and bones are the most common sites of metastasis [6]. There is a correlation between the stage of melanoma and the overall survival rate, with median survival durations varying significantly across stages: 138 months for stage I, 19 months for stage II, and 17 months for stage III [6]. AMM is often diagnosed at advanced stages, which contributes to its poor prognosis [6].

AMM is characterized by a high propensity for metastasis and nonspecific symptoms, which lead to poor prognosis. Around 20% of patients have lymph node-positive disease in the inguinal region, and systemic metastasis is detected in 7%-25% of cases at the time of diagnosis [5]. The most common sites for metastasis include the lungs, pelvis, and liver [11].

There is no correlation between AMM’s anatomical site (rectal, anorectal, or anal) and disease-free survival or overall survival [20]. Furthermore, there is no observed correlation between prognosis and factors such as sex, age, and ethnicity [21].

Prognostic factors include lymph node involvement, extent of spread, tumor necrosis, perineural invasion, and tumor thickness, with perineural invasion and tumor thickness being significant predictors of recurrence [13,21]. The Breslow index, which assesses lesion thickness, plays a crucial role in predicting prognosis. The thinner the lesion at removal, the better the prognosis [22]. Overall, the five-year survival rate for AMM is less than 20%, with a median survival of only two years, regardless of treatment modality [6]. This poor prognosis is attributed to delayed diagnosis, the inherent aggressiveness of the disease, and early lymphatic spread [6]. Therefore, any atypical anorectal lesion should be biopsied promptly to prevent delays in diagnosis [6].

A multidisciplinary tumor board should evaluate each patient’s case, and the treatment approach should be individualized based on factors such as tumor size, age, presence of comorbidities, and extent of metastases. The treatment options could involve surgery, chemotherapy, targeted therapy, immunotherapy, or radiation therapy based on the stage of the disease, aiming to prolong survival and improve quality of life. However, there are no consensus guidelines for AMM treatment due to its rarity and the lack of randomized clinical trials. Most treatment strategies are extrapolated from cutaneous melanoma guidelines.

The main therapy for localized disease is surgical resection, which may be done by either wide local excision (WLE) or abdominoperineal resection (APR) [5]. The current gold standard for surgical procedures has not been determined. Despite APR being more radical, studies have not shown improved survival compared to WLE, which is preferred when sphincter involvement is not a concern due to lower morbidity [23]. Thibault et al. reviewed 50 AMM cases and found no difference in five-year survival and recurrence rates between local excision and APR [24]. Considering that WLE is linked to lower morbidity and similar survival rates, it is logical to view it as the preferred first treatment option. There is a strong correlation between APR and significant morbidity and functional compromise [23]. Many surgeons perform WLE followed by adjuvant radiotherapy to achieve similar local control as APR [6].

Over the past decades, several therapies have been developed for AMM. Alpha interferon has demonstrated significant benefits in extending relapse-free survival and overall survival in patients with positive lymph nodes, although it is associated with considerable side effects, including hematological, autoimmune, and neuropsychiatric disorders [25].

There have been cases where patients remained cancer-free for over 10 months following neoadjuvant chemoradiotherapy or radiotherapy. However, the effectiveness of chemotherapy and radiotherapy is generally restricted due to the tumor’s typical resistance to these treatments [26].

Metastatic melanoma is often treated with interleukin-2 (IL-2), either alone or in combination with chemotherapy or biotherapy [6]. While combining IL-2 with chemotherapy and/or interferon boosts tumor response rates, it does not lead to better long-term survival outcomes [6]. Traditional chemotherapeutic agents such as dacarbazine and temozolomide are generally not very effective for metastatic mucosal melanoma [6]. However, dacarbazine has shown benefits in 10%-20% of patients with mucosal melanoma, whether administered alone or in combination with high-dose interferon and IL-2 [27].

Emerging immunotherapeutic strategies, such as immune checkpoint inhibitors, are demonstrating encouraging outcomes in the treatment of metastatic melanoma. Ipilimumab, an anti-CTLA4 antibody, has shown promising results, including a median overall survival of 4.3 months and a progression-free survival of 6.4 months [28]. Ipilimumab was the first immune checkpoint inhibitor approved for advanced melanoma [6]. Ongoing research is evaluating the efficacy of other immune checkpoint inhibitors, such as nivolumab and pembrolizumab, in treating mucosal melanoma [6].

Combination therapy with nivolumab and ipilimumab has improved progression-free survival in patients with advanced melanoma [29]. Metastatic melanomas with *KIT* mutations respond well to targeted therapy using specific inhibitors [30]. Favorable outcomes have been reported in patients with *KIT*-mutated rectal melanoma, and, in some cases, complete remission has been achieved in patients with *KIT*-mutated melanoma following treatment with sunitinib [31]. In general, the varied nature of mutations in AMM indicates that exploring the biology of tumors could uncover new target molecules that may have therapeutic benefits.

## Conclusions

AMM is a rare, aggressive tumor with a poor prognosis. It should always be considered in the differential diagnosis for patients presenting with anorectal complaints, particularly older adults. The lack of standardized treatment guidelines calls for a multidisciplinary approach tailored to each patient’s condition, considering tumor size, metastases, and overall health. The primary treatment for AMM is surgical resection with clear margins, either through WLE or APR. Neoadjuvant treatment, such as radiotherapy, may be considered in locally advanced cases to reduce tumor size and improve surgical outcomes. For advanced disease, palliative treatment may be needed. Emerging immunotherapies show promise but require further study.
